# Identification of* Lilium ledebourii* antiproliferative compounds against skin, bone and oral cancer cells

**DOI:** 10.22038/AJP.2023.22875

**Published:** 2023

**Authors:** Nastaran Partovi, Hassan Hassani Kumleh, Ebrahim Mirzajani, Mohsen Farhadpour

**Affiliations:** 1 *Department * *of* * Plant Biotechnology, Faculty of Agriculture, University of Guilan, Rasht, Iran*; 2 *Department of Biochemistry, Faculty of Medicine, Guilan University of Medical Sciences, Rasht, Iran*; 3 *Phytochemistry Group, National Institute of Genetic Engineering and Biotechnology, Tehran, Iran*

**Keywords:** Apoptosis, A431 cell line, KB cell line, G292 cell line, Phytochemicals

## Abstract

**Objective:**

This study aimed at the evaluation of anti antiproliferative activity of *Lonicera nummularifolia*, *Lilium ledebourii*, *Campsis radicans* and *Parthenocissus quinquefolia* extracts*. *

**Materials and Methods:**

The extract was taken from the fresh leaves and bulbs of the plants by maceration method in the dark. After separating the solvent, the remaining dry matter was added to the culture medium containing G292, A431 and KB cancer and HGF-1 normal cells. Cytotoxicity tests, as well as cell cycle and apoptosis tests were performed on cells treated with dry substances and untreated cells. Finally, the most effective extract was separated into fractions by preparative HPLC and the effective fraction was characterized by Triple-Quad LC/MS connected to the UHPLC system.

**Results:**

All extracts significantly enhanced cell death rate in the three cancer cell lines more than the HGF-1 line. The Methanolic extract of *L. ledebourii* bulbs exhibited considerable efficacy on apoptosis induction in the cancer cell lines. It seems that the mode of action for *L. ledebourii* methanolic extract is mediated through increased *BID/MAPK14* expression and decreased *MDM2/BCL2*/*MYC* expression, which led to activation of the p53 protein-induced apoptosis. It was also determined that the effective fraction of *L. ledebourii* methanolic extract consists of substances such as caffeic acid, ferulic acid, coumarin acid, catechin and apigenin.

**Conclusion:**

Overall, the findings suggest that *L. ledebourii* is a promising source of bioactive compounds with anticancer properties.

## Introduction

Cancer is one of the important causes of global death, thus, it challenges public health. The outbreak of this disease is rising, especially in South and Central America, Asia and Africa which makes up approximately 75% of deaths globally (Siegel et al., 2020 ▶). Considerable research has been focused on the development of different agents for tumor therapy. The progress in chemical anticancer drugs development has improved patients’ health and thereby chemotherapy has appeared as one of the potential options to treat a wide range of tumors (Pucci et al., 2019 ▶). But, these chemicals in turn lead to adverse side effects on human tissues/cells, such as alopecia, vomiting, nausea and bone marrow function inhibition (Chikara et al., 2018 ▶). In contrast, many phytochemicals and natural compounds have been proven as anticancer adjuvant therapy because of their proapoptotic, antioxidative, and antiproliferative features (Seca and Pinto, 2018 ▶). Accordingly, the continuing research for discovery of anticancer medicines from herbs plays an important role to determine the feasible options to have safeness and to decline the side effects induced by chemical drugs since natural phytochemicals have some promising benefits (Greenwell and Rahman, 2015 ▶).

From previous reports, most biologically active compounds act as anticancer agents via their potential to induce apoptosis (D'Arcy, 2019 ▶). Programmed cell death as a potential target for tumor therapy plays a crucial role in the establishment, homeostasis, and removal of any abnormal cells by using a variety of cellular signaling pathways (Elmore, 2007 ▶). Since apoptosis repression in carcinogenesis can disturb the balance between cell death and proliferation, it leads to the establishment and progression of cancers (D'Arcy, 2019 ▶). Albeit apoptosis induction in tumor cells is an interesting remedy for tumor chemotherapy, the variety of molecular mechanisms in such abnormal cells has complicated the analysis of the results (Renehan et al., 2001 ▶).

Numerous therapeutic properties have been reported for *Lonicera nummularifolia*, *Lilium ledebourii*, *Campsis radicans* and *Parthenocissus quinquefolia*. For instance, Farboodniay Jahromi et al. (2020) ▶ demonstrated the high antimicrobial property and ferric-reducing activity along with free radical scavenging in *L. nummularifolia* (Farboodniay Jahromi et al., 2020 ▶). Islam et al. (2019) ▶ presented evidence on the hypoglycemic, analgesic, membrane stabilizing, thrombolytic and antioxidant activities of *C. radicans* (Islam et al., 2019 ▶). Faisal et al. (2018) ▶ recorded the antioxidant activity of *P. quinquefolia* due to alkaloids, flavonoids and terpenoids (Faisal et al., 2018 ▶). Shokrollahi et al. (2018) ▶ indicated that the antioxidant capacity of *L. ledebourii* is relatively high compared to other medicinal herbs (Shokrollahi et al., 2018 ▶). Despite such studies, there is a lack of information on the cytotoxicity of all medicinal herbs and their mechanism of action on cancer cells. In the current research, we assessed the cytotoxic efficacy of extracts derived from four plants, including *L. nummularifolia, L. ledebourii, C. radicans *and* P. quinquefolia*, on some cancer cells including oral squamous cell carcinoma and osteosarcoma (KB cell line), skin cancer (A431 cell line) and bone cancer (G292 cell line) in comparison to HGF-1 as a normal cell line. 

## Materials and Methods

**Plant material and extract preparation**

The leaves of three herbs including *L. nummularifolia *(voucher number: CAL4225)*, C. radicans *(voucher number: BIC2267) and* P. quinquefolia* (voucher number: VIP2246) along with the bulbs of *L. ledebourii *(voucher number: LIL1228) were collected from Guilan forests, northern Iran. Then, 100 g of each sample was washed three times by sterile distilled water. Their extraction was done via pure methanol and ethanol (80%) in a volume of 350 ml through maceration method at 50 rpm, at 25°C for 24 hr in darkness (Rezadoost et al., 2019a ▶). Each extract was filtered using Whatman® Grade 42 Ashless Filter Papers (Sigma-Aldrich) and then dried via a freeze dryer. Then, 1 mg of resulted material was solved in 1 ml of 0.1% (w/v) dimethyl sulfoxide (DMSO) through the medium RPMI to prepare the stock solution. Eventually, the resulting extracts were incorporated into the cell culture media.

**Cell culture**

KB, A431 and G292 cell lines along with HGF-1 as a normal cell line were provided by the Biological Resource Center, Iran. These cell lines were grown in RPMI-1640 medium containing 1% (w/v) streptomycin-penicillin antibiotics and 10% (w/v) fetal bovine serum (Sigma-Aldrich) and they were kept in an incubator under 5% CO_2_ at 37°C (Ackermann and Tardito, 2019 ▶).

**MTT assay**

First, the intended cell lines were trypsinized and counted via Neubauer Chamber. Then, 7×10^3^ cells were cultured in 100 μl of RPMI-1640 medium containing 10% (w/v) fetal bovine serum and 1% (w/v) streptomycin-penicillin antibiotics in a 96-well microplate and incubated for 24 hr at 37°C under 5% CO_2_. To achieve a sufficient number of cells per plate, the cells were permitted to adhere to the plate bottom and then, supernatants were removed. The 50 μg/ml (dissolved in RPMI-1640 medium containing 1% (w/v) streptomycin-penicillin antibiotics and 10% (w/v) fetal bovine serum) of each herb extract was incorporated into each well. Control cells of each line were treated only with 0.1% (w/v) DMSO. Following 24 hr, the culture medium was removed and 20 μl of fetal bovine serum including 0.2 mg/ml of MTT (3-(4, 5-dimethylthiazol-2-yl)-2, 5-diphenyl-2H tetrazolium bromide) reagent was incorporated into cells. Eventually, the insoluble formazan crystals were dissolved in 0.1% DMSO and the absorbance was read via a spectrophotometer at 570 nm (Florento et al., 2012 ▶).

**Flow cytometry**

Flow cytometry technique was utilized to assay the cell cycle and apoptosis in cancer cells. For achieving this goal, 50 μg/ml of each cell line was collected overnight and homogenized in 0.5 ml of phosphate-buffered saline by vortexing.

The 70% ethanol (1 ml) was mixed with the solution and maintained on ice for 120 min. The resulting mixtures were centrifuged for 15 min at 300 ×g, and then supernatants were discarded. Then, 5 mL of phosphate-buffered saline was incorporated into each pellet for 30 sec and the mixture was centrifuged for 5 min at 300 ×g. After the supernatant was discarded, the residue was utilized to evaluate the cell cycle and apoptosis using the Annexin V -propidium iodide (PI) mixture along with a PI solution (according to the manufacturer’s instruction, Sigma kit). After 30 min, the mixtures were injected into the flow cytometry instrument (Gupta et al., 2011 ▶).

**RNA extraction and qRT-PCR**

The gene expression assay was accomplished on a total of 10^6^ cells treated with the IC25 (inhabitation concentration) of doxorubicin and *L. ledebourii* methanolic extract for 24 hr. Total RNA from the cells was extracted via RNX-Plus solution (CinnaGen, Iran) based on the manufacturer’s instructions. The First Strand cDNA Synthesis Kit was utilized to synthesize cDNA from RNA (Thermo Fisher Scientific). Primer design for MYC proto-oncogene, bHLH transcription factor (MYC; ID), p53 (P53; ID), BH3 interacting-domain death agonist (BID; ID), Map Kinase 14 (MAPK14; ID), MDM2 proto-oncogene (MDM2; NM_001145337) and B-cell lymphoma 2 (BCL2; NM_000633) was accomplished based on NCBI database and via primer3 software (Table 1.). qRT-PCR was run through SYBR Green PCR MasterMix (Thermo Fisher Scientific) in 40 cycles, including denaturation for the 60 sec at 95°C, annealing for 20 sec at 55-60°C and extension for 20 sec at 72°C. *GAPDH* (NM_001256799) was used as a reference gene to estimate the steady-state transcript level of each gene. The 2^−ΔΔCt ^method was followed to calculate the fold change (Livak and Schmittgen, 2001 ▶). 

**Table 1:** Primers utilized for qRT-PCR

**Gene name**	**Primers**	**Tm°C**
***MYC***	5’-GGAACTTACAACACCCGAGC-3’ 5’-GCTGCCATCACTGTTAAGCT-3’	56
***P53***	5’-TGGCCATCTACAAGCAGTCA-3’ 5’-GGTACAGTCAGAGCCAACCT-3’	56
***BID***	5’-CCTACTGGTGTTTGGCTTCC-3’ 5’-GCCTCTATTCTTCCCAAGCG-3’	56
***MAPK14***	5’-CTGGATTTTGGACTGGCTCG-3’ 5’-AGTCAACAGCTCGGCCATTA-3’	57
***MDM2***	5’-TCACAGATTCCAGCTTCGGA-3’ 5’-GCACGCCAAACAAATCTCCT-3’	56
***BCL2***	5’-TTCTTTGAGTTCGGTGGGGT-3’ 5’-CTTCAGAGACAGCCAGGAGA-3’	56
***GAPDH***	5’-TCACCAGGGCTGCTTTTAAC-3’ 5’-GGACTCCACGACGTACTCAG-3’	56

**Instrumental analysis**

UHPLC system was used for separating the extracts’ constituents. This system includes an autosampler, a binary pump and a vacuum degasser. It consists of a reversed-phase rapid resolution C18 analytical column of 50 mm × 4.6 mm i.d. and 1.8 µm particle size (RR Zorbax Eclipse XDB-C18). For each sample, there was a 10 µL injection of the extract. The elution was conducted in gradient mode as follows: water with 0.1% formic acid served as eluent A and acetonitrile was used as eluent B. Using the chromatographic technique, the primary mobile phase composition (10% eluent B) constant was kept for one minute and then a linear gradient followed to 100% eluent B in11 minutes. Then, 100% eluent B was passed through the column for four minutes. The overall run time was 20 min, with 0.6 ml/min flow rate.

The crude extract was separated by preparative-HPLC into 9 fractions (C1-C9) based on peak and retention time at different time points including 21 (C1), 30.5 (C2), 35.5 (C3), 38(C4), 40 (C5) 41.5 (C6), 50.5 (C7), 48 (C8), 60 (C9) min. The effective ingredient was identified by mass spectrometry.

**Triple-Quad LC/MS analysis**

A triple quadrupole mass spectrometer Agilent 6410 Triple-Quad LC/MS which was connected to the UHPLC system, was used as the detector. The spectrometer that was consisted of an electrospray interface working in positive ion mode, used these operating factors: nebulizer gas: 50 psi, capillary voltage: 5000 V, gas temperature: 325°C, gas flow: 12 L/min. Nitrogen was used as the collision into the ESI (Electrospray ionization) source in positive mode and specific MRM (Multiple reaction monitoring) transition, collision energy and fragment voltage were optimized. Agilent Mass Hunter Software was employed to develop the method and data acquisition and processing, including MRM Mode Software and Mass Hunter Optimizer feature.

**Statistical analysis**

Doxorubicin was used as a standard drug and compared to the herb extracts. IC50 for each extract was calculated through GraphPad Prism 7. All experiments were performed in a completely randomized factorial design with three replicates. Data analyses were done using SPSS 20 (SPSS Inc., Chicago, IL, USA). Data are presented as mean values±standard error of the meaning (SE). One-way analysis of variance test (ANOVA) was used for statistical analysis of data and Duncan's *post hoc* test was used to compare the means of the groups. The differences were considered to be statistically significant at p<0.05.

## Results

**Cytotoxicity of the herb extracts**

**Figure 1:**
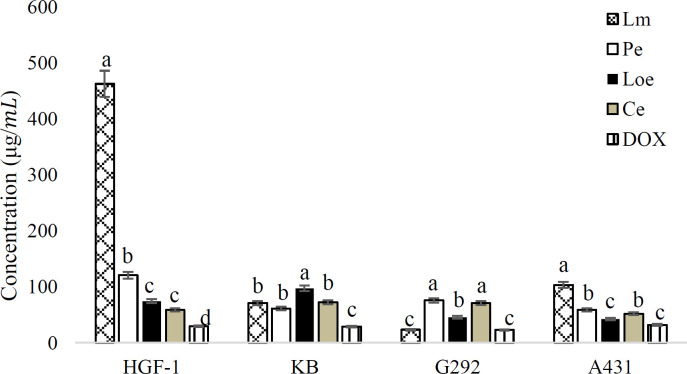
The IC50 value of doxorubicin and the herb extracts against the cancer (G292, A431 and KB) and normal (HGF-1) cell lines (Lm: Methanolic extract of *L. ledebourii* bulbs; Pe: Ethanolic extract of *P. quinquefolia* leaves; Loe: Ethanolic extract of *L. nummularifolia* leaves; Ce: Ethanolic extract of *C. radicans* leaves; DOX: doxorubicin. The data are expressed as mean±SEM. The letters indicate significant differences of means at p≤0.05)

The cytotoxicity rate was compared between HGF-1 and cancer cell lines because of two reasons: i) the increasing concentration of the herb extracts changed the toxicity rate in a dose-dependent way and ii) the decrease of toxicity in a normal cell line which is considered an optimum safety index. From the results, the IC50 value of the methanolic extract of *L. ledebourii* bulbs (Lm) showed the highest cytotoxicity in the G292 cell line and less cytotoxicity in the HGF-1 line. In spite of the higher cytotoxicity of other extracts compared to the Lm extract on the A431 line, this extract was superior because of lower toxicity in the HGF-1 line (Figure 1b).

**Apoptosis induction by herb extracts**

**Figure 2:**
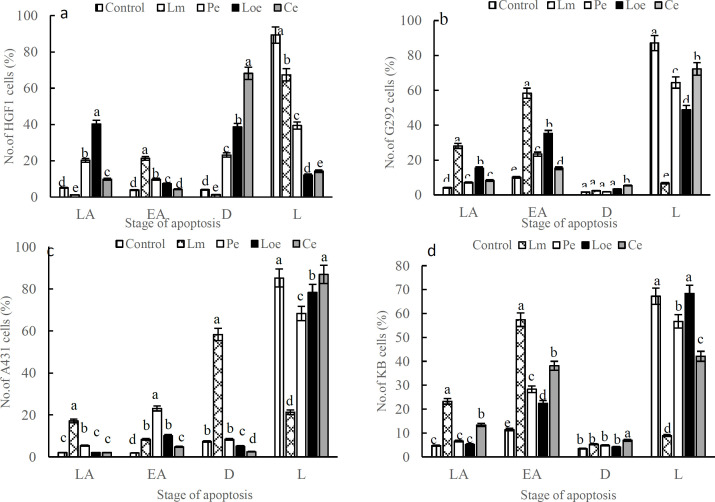
Flow cytometry of apoptosis in the G292, A431 and KB cancer lines along with HGF-1 normal cell line by 50 μg mL^−1^ of the herb extracts (The percentage of cells in various steps of apoptosis was tagged with D: dead, L: live, LA: late apoptosis, EA: early apoptosis. Lm: Methanolic extract of *L. ledebourii* bulbs; Pe: Ethanolic extract of *P. quinquefolia* leaves; Loe: Ethanolic extract of *L. nummularifolia* leaves; Ce: Ethanolic extract of *C. radicans* leaves. The data are expressed as mean±SEM. The letters indicate significant differences of means at p≤0.05)

All extracts significantly decreased the number of alive HGF-1 cells over the control. The maximum number of alive cells (67.4%) in the HGF-1 line was recorded from the Lm extract. This extract increased the number of early apoptosis (EA) cells more than other extracts in the HGF-1 line. Because the ethanolic extract of *L. nummularifolia* leaf (Le)-treated HGF-1 line presented a high percentage (40.3%) of LA (late apoptosis) cells when compared to Lm extract, which was ~0%, thus it displays a non-selective feature of apoptosis for Loe. Besides, the Ethanolic extract of *C. radicans* leaves (Ce) significantly increased the number of dead cells in the HGF-1 line compared to the control, which indicates the non-apoptotic lethal effect of this extract (Figure 2a).

**Figure S1:**
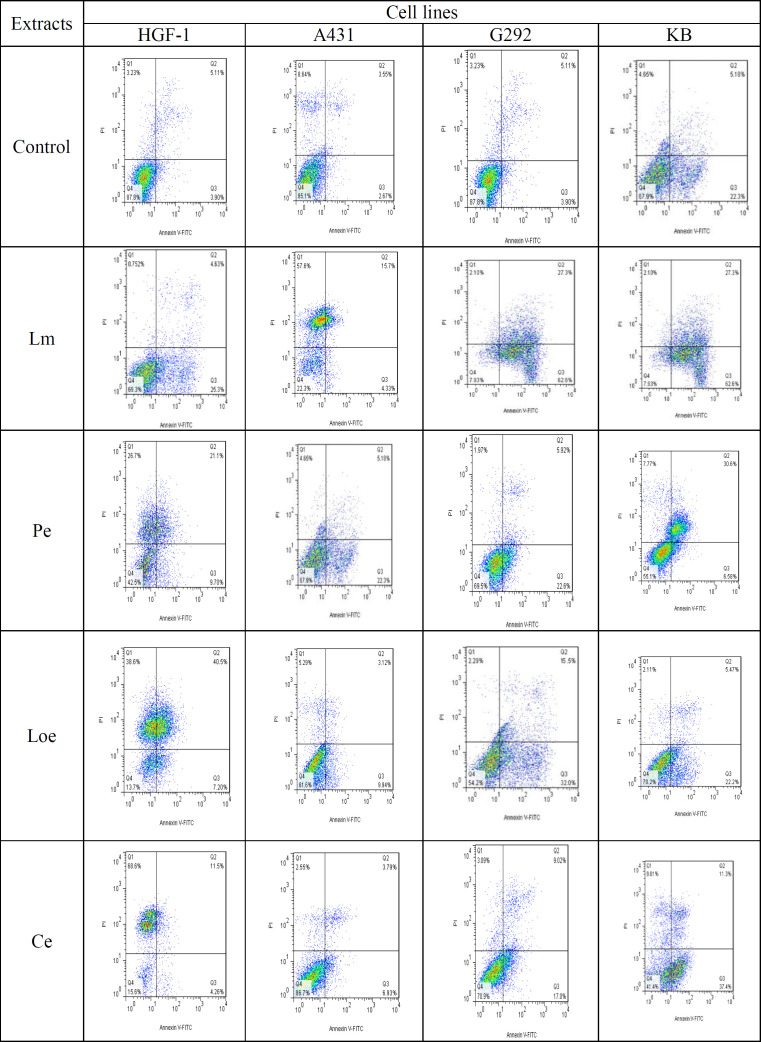
Analysis of apoptosis and cell cycle by flow cytometry in treated and non-treated cancer cells (G292, A431, and KB) and normal (HGF-1) cells with extracts. For apoptosis, the cells were stained with annexin V PE and ReadiDrop Propidium Iodide. Apoptotic cells positive for annexin V can be seen in the bottom right quadrant and dead cells positive for both annexin and PI in the top right quadrant. Healthy cells are negative for both stains. Lm: Methanolic extract of *L. ledebourii* bulbs; Pe: Ethanolic extract of *P. quinquefolia* leaves; Loe: Ethanolic extract of *L. nummularifolia* leaves; Ce: Ethanolic extract of *C. radicans* leaves.

In the G292 line, albeit four herb extracts significantly enhanced the EA cells over the control, the Lm extract demonstrated a maximum rate of LA cells than the control. Moreover, it seems that the lethal efficacy of Lm on the G292 cell line is associated with necrosis, not apoptosis (Figure 2b). In the A431 line, the maximum rate of EA cells was observed in the lines treated with the ethanolic extract of *P. quinquefolia* leaves (Pe) whereas the Lm extract significantly increased the rate of LA cells and dead cells than the control and other herb extracts. The high death of the A431 cell line treated with Lm extract (58.3%) suggested necrotic death (Figure 2c). In the KB line, the Lm extract demonstrated the highest toxicity by EA and LA cells and the lowest rate of living cells (8.9%), while the Ce extract displayed the least toxicity among all extracts. In evaluating early apoptosis, the Lm extract exhibited the highest level with a striking percentage of 47.4%, followed by Ce with 28.1% while the Loe extracts derived the lowest rate of early apoptosis among all herb extracts (Figure 2d). additional data are given in Online Resource 1 (Figure S1).

**Cell cycle assay**

**Figure 3:**
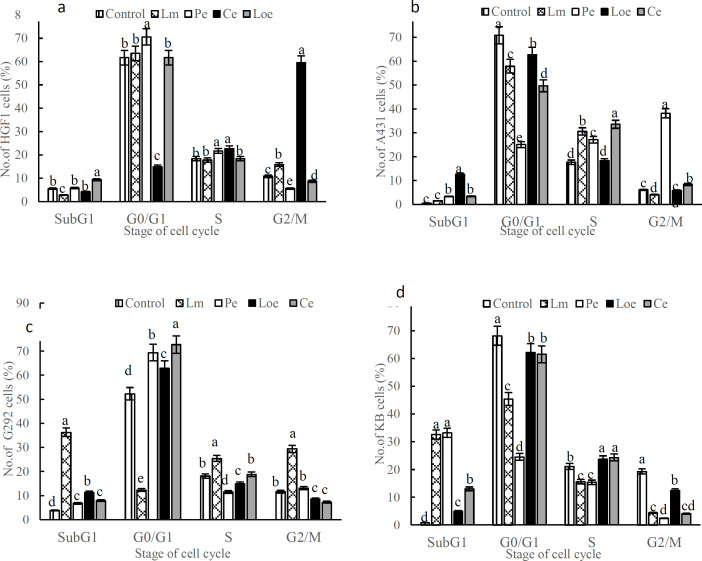
Flow cytometry of cell cycle stages in the G292, A431 and KB cancer lines along with HGF-1 normal cell line by 50 μg mL^−1^ of each herb extract (Lm: Methanolic extract of *L. ledebourii* bulbs; Pe: Ethanolic extract of *P. quinquefolia* leaves; Loe: Ethanolic extract of *L. nummularifolia* leaves; Ce: Ethanolic extract of *C. radicans* leaves. The data are expressed as mean±SEM. The letters indicate significant differences of means at p≤0.05)

As demonstrated in Figure 3c, the methanolic extract of *L. ledebourii* bulbs (Lm) exhibited the highest accumulation of cells in the subG1 step of G292 and KB lines compared to the control. This extract resulted in the highest rate of apoptosis in G292 with a remarkable quantity of 36.3% of total cells in the subG1 step over control. Besides that, the ethanolic extract of *P. quinquefolia* leaves (Pe) significantly enhanced the number of cells in the subG1 in KB line compared to the control (Figure 3d). However, both of these herb extracts (Lm and Pe) displayed a very slight efficacy on the HGF-1 and A431 lines and their entrance into the subG1 step. In the presence of the Ce extract, although the number of sub-G1 cells in all cancer lines was more than the control, the number of the HGF-1 cells treated with this extract was more than in other treatments just in the G2 step (Figure 3a). 

**Figure S2:**
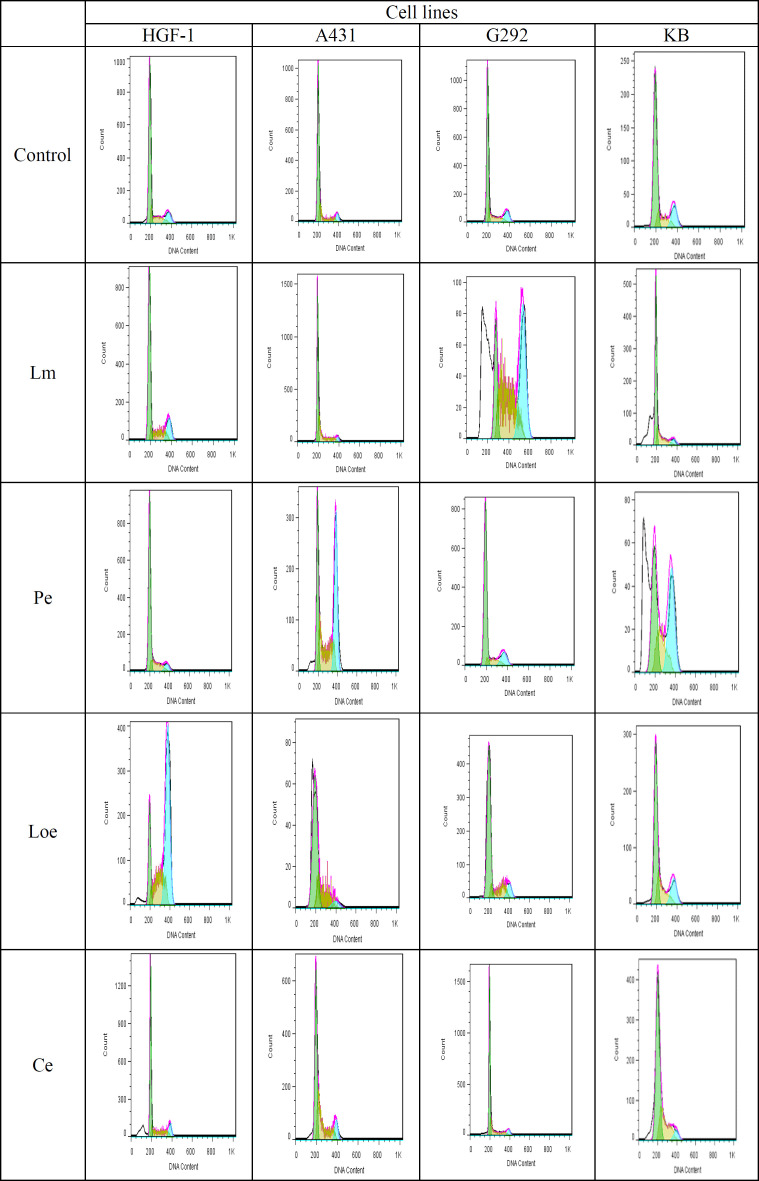
Cell cycle analysis by flow cytometry on cancer (MCF-7, A431 and U87-MG) and normal (HGF-1) cell lines. Different phases of cell cycle were determined. Lm: Methanolic extract of *L. ledebourii* bulbs; Pe: Ethanolic extract of *P. quinquefolia* leaves; Loe: Ethanolic extract of *L. nummularifolia* leaves; Ce: Ethanolic extract of *C. radicans* leaves.

Moreover, the Lm and Loe extracts could increase the number of cells that had been stopped in the S phase in all the cancer cells (Figure 3a–d). From our observations, the apoptosis, cell cycle, and MTT tests revealed that the Lm extract plays a vital role in the death acceleration of tumor cells. Additional data are given in the Online Resource 2 (Figure S2).

**The expression of genes involved in apoptosis**

**Figure 4:**
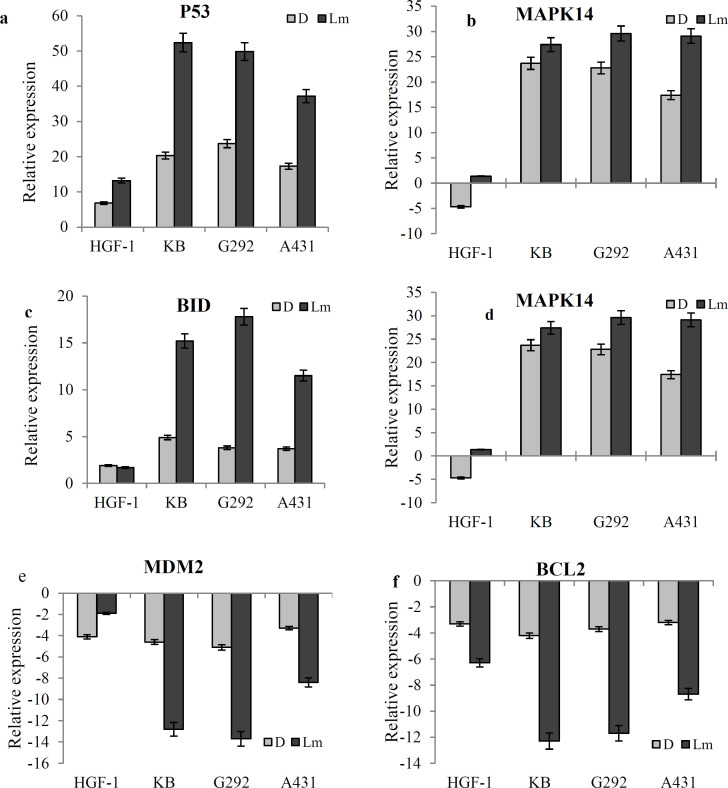
Expression of *MYC*, *P53*, *BID*, *MAPK14*, *MDM2* and *BCL2* genes in response to the *L. ledebourii* methanolic extract (Lm) and doxorubicin (D) in the cancer (G292, A431 and KB) and normal (HGF-1) cell lines. The data are expressed as mean±SEM (The letters indicate significant differences of means at p≤0.05)

*L. ledebourii *extract exhibited a significant change in the transcript level of some genes in cancer cells. However, the expression patterns of other genes were similar to doxorubicin. The transcript level of *P53, BID, and MAPK14* genes enhanced significantly compared to doxorubicin (Figure 4a-c). In contrast to the* L. ledebourii *extract, doxorubicin significantly induced the transcript level of the *MYC *in the tumor and normal cell lines (Figure 4d). The transcript level of *MDM2 *and *BCL2 *apoptotic inhibitors genes was significantly decreased in response to Lm extract compared to doxorubicin (Figure 4e-f

**Characterization of antiproliferative compounds**

**Figure 5:**
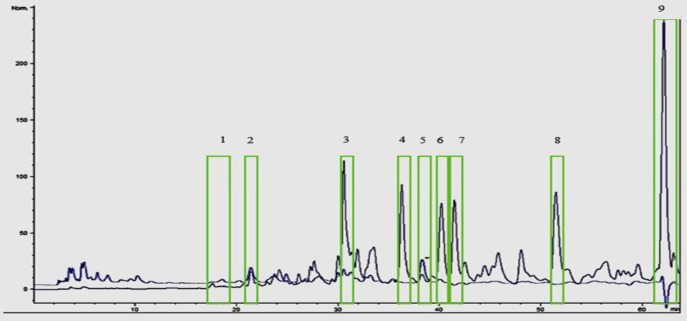
Analytical chromatogram for injection of methanolic extract of *L. ledebourii* bulbs at two wavelengths 270 and 305 nm

**Figure 6:**
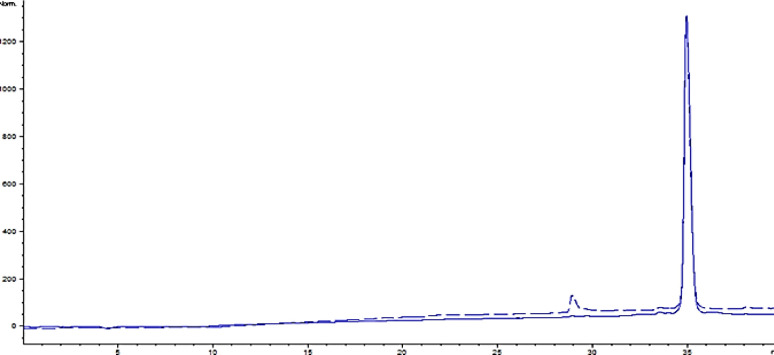
Analytical chromatogram for injection of fraction 8 at wavelength 305 nm

**Figure 7:**
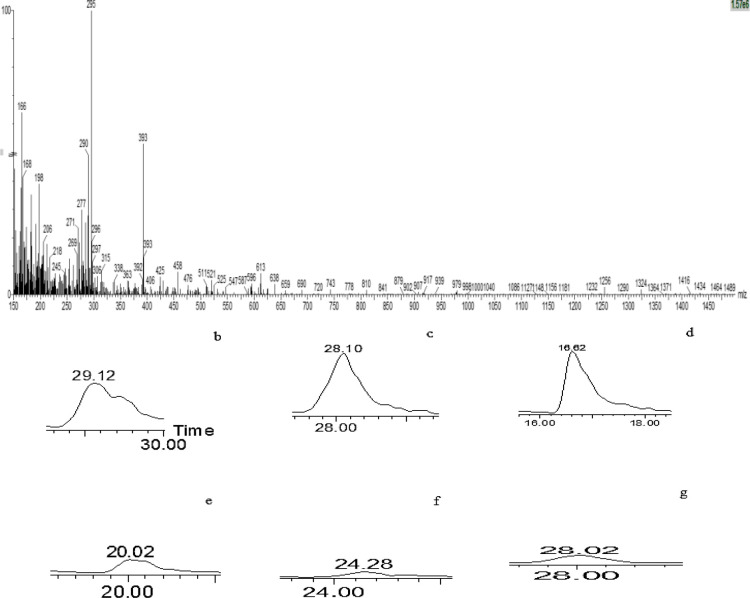
Mass spectrum chromatogram for the Fraction 8 obtained from *L. ledebourii* methanolic extract (a), Mass spectrum data chromatogram related to (b) catechin, (c) coumarin acid, (d) caffeic acid, (e) ferulic acid, (f) kaempferol, (g) apigenin

To select the optimum extract with a high level of lethal efficiency on cancer cells, several factors were considered including IC50, cell number in sub-G1 and scatter gating of cell population in histograms of Annexin V and PI. Therefore, the methanolic extract of *L. ledebourii bulbs, *included a balanced of all these factors, almost indicated the high cytotoxicity level in cancer cell lines and was selected as a significant anticancer agent. After separating the fractions of the extract by preparative-HPLC, in accordance with the chromatogram provided in Figure 5, the most effective fraction was identified by examining the toxic effects of each fraction on G292 cell line. The results have shown that the 8th fraction among 9 fractions had the highest toxicity on cell line G292 (Table 2). In order to investigate the purity of fraction 8, analytical chromatography was used when its chromatogram is according to Figure 6. The results have shown that the purity of the purified compound was high to identify and determine the structure. The results of identifying the sample structure of fraction 8 and matching of m/z results with similar articles showed that it was mainly composed of coumarin acid, catechin, caffeic and ferulic acid, kaempferol and apigenin which is connected by one molecule of glycerol, and the glycerol group are formed by para/ortho substitution or glucose substitution/acetylation (Munafo and Gianfagna, 2015). Therefore, these compounds belong to phenolic glycerides/glycosides (phenylpropanoid compounds) and are called “regalosides” (Table 3 and Figure 7a). The mass spectrum chromatogram for the identified materials is presented in Figure 7b.

**Table 2:** Percentages of cancer (G292) and normal (HGF-1) cell death (cell toxicity) induced by different fractions of *L. ledebourii* after 24 hr Treatment at 50 μg/ml

Fraction	Cell lines (Mean±SE)	
	G292	HGF-1
Lm1	8.15	7.52
Lm2	12.87	8.36
Lm3	29.34	11.25
Lm4	43.16	13.62
Lm5	52.13	14.91
Lm6	56.43	17.32
Lm7	67.24	18.75
Lm8	94.23*	23.37
Lm9	74.65	18.14

(The * indicate significant difference of means at p≤0.05).

**Table 3:** List of major phenolic compounds identified by Triple-Quad LC/MS in the Fraction 8 obtained from *L. ledebourii* methanolic extract

Compounds	m/z	Reference
Caffeic acid	166-329-667-511	Zhao et al., 2021 ▶; Ben Said et al., 2017
Ferulic acid	200	
Coumarin acid	295-277-365-205-269-541-280	Zhao et al., 2021 ▶; Ben Said et al., 2017
Catechin	290-289-271-247-245-306-205-458-425-578-	Pe´rez-Magarino et al., 1999; Ben Said et al., 2017
Kaempferol	241	Pe´rez-Magarino., 1999
Apigenin	269-271	Tsimogiannis et al., 2007; Bouaziz et al., 2005

## Discussion

Based on the MTT assay, four extracts significantly enhanced cell death in the cancer cell lines, compared to the HGF-1 cell line. The high toxicity levels in the cancer cell lines were recorded for Loe on G292 and A431, Pe on KB and A431, Ce on A431, as well as Lm on G292 and KB. Similarly, Farboodniay-Jahromi et al. (2020) ▶ demonstrated that alkaloids, flavonoids, and phenols can justify clearly the biological activity of *L. nummularifolia* on the cell lines (Farboodniay Jahromi et al., 2020 ▶).

As our observations revealed, the methanolic extract of *L. ledebourii* bulbs (Lm) could induce programmed cell death and enhance cell accumulation in the subG1 step and the rest of the cell cycle in the G0/G1 step. *L. ledebourii *has been proven as a herb, containing lectin as a carbohydrate-binding protein (Mahdinezhad et al., 2018 ▶). Several researches demonstrated the anticancer efficacy of these proteins in apoptosis induction, cell accumulation in G0/G1 and/or G2/M phases and ribosomal attachment (Mishra et al., 2019 ▶).

From previous reports, it was well-known that the *BH3 interacting-domain death agonist (BID) *gene is up-regulated via the *p53* tumor suppressor and it is involved in *p53*-induced apoptosis (Mantovani et al., 2019 ▶). Moreover, apoptotic motivators induce *caspase8* and its substrate, *BH3* interacting-domain death agonist, in a death receptor-independent way (Mantovani et al., 2019 ▶). Our observations are in the line with these reports, suggesting that the *L. ledebourii* extract can increase *BID* and subsequent *P53* gene expression, thereby it helps to induce apoptosis in the KB, A431 and G292 cell lines. 

*Map Kinase 14*, a crucial member of the MAPK family *P38*, plays a dual function in some tumors (Chen et al., 2018 ▶). Several researchers have indicated that *Map Kinase 14* can promote the onset and progress of breast tumors by inducing its downstream genes (Phesse et al., 2014 ▶). With the given inhibitory role of *MAPK14* in skin cancer cell line (Rezadoost et al., 2019b ▶), it seems that the increased level of *MAPK14* transcript derived by Loe extract can be effective in controlling cell growth through apoptosis.

Cancer cells usually enhance the *MYC* gene expression as a consequence of tonic WNT signaling, *MYC* gene amplification and/or chromosomal translocation (Gupta et al., 2014 ▶). By gain of prosurvival signals (e.g., NF-κB and *BCL2*) and/or by loss of surveillance mechanism (e.g., *MDM2* and *P53)*, cancer cells can tolerate the enhanced *MYC *level and thereby avoid programmed cell death (Wagner and Nebreda, 2009 ▶). As proved in this study, the *L. ledebourii* methanolic extract decreased the transcript level of *MYC*. Thus, it seems that *L. ledebourii* induces negative auto-regulation which decreases *MYC* expression in turn.

It is well-known that the *MDM2/BCL2* dual repression makes an opportunity for a bioactive compound to act as an antitumor agent (Billen et al., 2008 ▶; Dhillon et al., 2007 ▶). These statements are in line with our findings when the *L. ledebourii* methanolic declined the transcript levels of *BCL2* and *MDM2*. Evidence of the active role of Le extract in increasing *p53* gene expression has been provided. Since the induction of *p53* tumor suppressor indirectly decreases several cell cycle genes and eventually results in the cell cycle arrest (Anwar et al., 2018 ▶), the *L. ledebourii* extract regulated a plethora of genes involved in the cell cycle by *MDM2/BCL2* and *p53* pathways and can contribute to cell cycle arrest. 

Based on the results of Triple-Quad LC/MS connected to the UHPLC analysis for *L. ledebourii *methanolic extract, it was determined that anticancer agent is a phenolic compound. The phenolic compounds are the secondary metabolites of plants, which have a wide range of biological activities, such as the anti-inflammatory, antioxidant, anti-aging, and antidepressant properties (Zhao et al., 2021 ▶). It has also been found that plant phenolic compounds can be effective in preventing the growth of cancer cells, their angiogenesis, and the production of metastatic cells (Wahle et al., 2010 ▶). According to the report of Luo et al. (Luo et al., 2012 ▶), the phenolic compounds in *L. brownii* are mainly phenylpropanoids, which have significant antioxidant activity.

As shown in this study, the methanolic extract of *L. ledebourii* significantly enhanced the cell death rate in G292, A431 and the KB cancer cells, compared to the HGF-1 cell line. Moreover, this herb extract can be a novel medicine for cancer therapy because of the high level of apoptosis activation in tumor cells and low cytotoxicity in normal cells. It seems that the mechanism of action for *L. ledebourii* methanolic extract is modulating *BID/MAPK14* along with *MDM2/BCL2/MYC* expression, which in turn, leads to the *P53* protein-induced apoptosis. 

## Conflicts of interest

The authors have declared that there is no conflict of interest.

## Supplementary
